# Predict initial subthalamic nucleus stimulation outcome in Parkinson's disease with brain morphology

**DOI:** 10.1111/cns.13797

**Published:** 2022-01-20

**Authors:** Yingchuan Chen, Guanyu Zhu, Yuye Liu, Defeng Liu, Tianshuo Yuan, Xin Zhang, Yin Jiang, Tingting Du, Jianguo Zhang

**Affiliations:** ^1^ Department of Neurosurgery Beijing Tiantan Hospital Capital Medical University Beijing China; ^2^ Department of Functional Neurosurgery Beijing Neurosurgical Institute Capital Medical University Beijing China; ^3^ Beijing Key Laboratory of Neurostimulation Beijing China

**Keywords:** brain morphology, efficacy, machine learning, subthalamic nucleus deep brain stimulation

## Abstract

**Aim:**

Subthalamic nucleus deep brain stimulation (STN‐DBS) has been reported to be effective in treating motor symptoms in Parkinson's disease (PD), which may be attributed to changes in the brain network. However, the association between brain morphology and initial STN‐DBS efficacy, as well as the performance of prediction using neuroimaging, has not been well illustrated. Therefore, we aim to investigate these issues.

**Methods:**

In the present study, 94 PD patients underwent bilateral STN‐DBS, and the initial stimulation efficacy was evaluated. Brain morphology was examined by magnetic resonance imaging (MRI). The volume of tissue activated in the motor STN was measured with MRI and computed tomography. The prediction of stimulation efficacy was achieved with a support vector machine, using brain morphology and other features, after feature selection and hyperparameter optimization.

**Results:**

A higher stimulation efficacy was correlated with a thicker right precentral cortex. No association with subcortical gray or white matter volumes was observed. These morphological features could estimate the individual stimulation response with an *r* value of 0.5678, an *R* ^2^ of 0.3224, and an average error of 11.4%. The permutation test suggested these predictions were not based on chance.

**Conclusion:**

Our results indicate that changes in morphology are associated with the initial stimulation motor response and could be used to predict individual initial stimulation‐related motor responses.

## Graphical abstract

The changes in morphology are associated with the initial stimulation motor response of subthalamic nuclei deep brain stimulation and could be used to predict individual initial stimulation‐related motor responses.

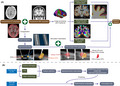

## 1 INTRODUCTION

Parkinson's disease (PD), the second most common neurodegenerative disease, has a great impairment on patients' motor and nonmotor skills.^1^ After an initial “honeymoon” period, during which medication can be effective, beneficial effects of medication are hampered by motor complications and progressive motor impairment.^2^ Fortunately, subthalamic nucleus deep brain stimulation (STN‐DBS), a powerful neuromodulation therapy, has been reported to be effective in treating motor symptoms, such as bradykinesia, rigidity, and tremor, in advanced^3^, ^4^ as well as early‐stage PD patients.^2^

Destruction of dopaminergic neurons in the substantia nigra pars compacta is the hallmark of PD. This phenomenon has been confirmed to be associated with alpha‐synuclein.^5^ According to the neuropathological Braak stages, alpha‐synuclein pathology ascends from the brainstem to the neocortex, which may result in changes in brain morphology and function.^6^ Cortical thinning of the left medial supplementary motor area (SMA) and in the right dorsal pre‐SMA has been observed in PD patients, and left temporal pole thickness has been correlated with disease duration.^7^ Furthermore, changes in subcortical volume have been observed in PD patients.^8^ Moreover, previous studies confirmed the abnormal functional connectivity and fiber projection in PD patients by functional magnetic resonance imaging (fMRI) and diffusion tensor imaging (DTI).^9^, ^10^

Despite significant effectiveness, STN‐DBS is an expensive and complicated surgery, and hence, the prediction of treatment efficacy is an important issue. Considering the changes in brain morphology and function, these features may be applied to predict the efficacy of this neuromodulation therapy. Recently, some researchers attempted to predict the efficacy of STN‐DBS on the motor response on the basis of fMRI and DTI. Horn et al. found that structural and functional connectivity were independent predictors of clinical improvement (based on the Unified Parkinson Disease Rating Scale [UPDRS]), with an average error of 15%.^11^ In another study, the volume of tissue activated (VTA) in the motor STN was shown to be able to associated with connectivity changes within the motor network across PD patients, indicating it is a potential predictor of DBS efficacy.^12^ Adaptive DBS (aDBS) has been reported to be more powerful and effective in controlling motor symptoms.^13^ The prediction of initial DBS efficacy could promote the development of aDBS to some degree. However, previous studies merely focused on the chronic efficacy of STN‐DBS in PD patients,^11^, ^12^ and the performance of initial efficacy predictions remains to be further investigated. Moreover, prediction of the initial response is beneficial to the prediction of the long‐term response, due to the relationship between these issues.^14^ For some PD patients with severe tremor and dyskinesia, it is difficult to undergo fMRI and DTI due to the long scan time and other requirements. Hence, brain morphology may be a more practical and universalizable predictor of DBS efficacy. It remains incomplete understand whether brain morphology is associated with DBS efficacy and whether these features can be applied to predict DBS efficacy.

In the present study, brain morphology in PD patients who underwent STN‐DBS was characterized by structural MRI, and its association with initial DBS efficacy was investigated. Subsequently, these features were used to predict initial STN‐DBS efficacy in the test set with a machine learning algorithm. Furthermore, the performances of different feature sets were compared. As expected, pathological changes in brain morphology were associated with and could be used to predict initial DBS efficacy, which may further the application and development of DBS.

## 2 MATERIAL AND METHODS

### 2.1 Patients and neurophysiological evaluations

A total of 94 patients with PD who underwent STN‐DBS between 2018 and 2020 were retrospectively enrolled in the present study. Patients were diagnosed with PD on the basis of the UK Brain Bank criteria.^15^ Clinical variables, including sex, age, disease duration before surgery, Hoehn‐Yahr (H‐Y) stage, and levodopa equivalent dose (LEDD, based on a previous study^16^), were collected. The Movement Disorder Society (MDS)‐UPDRS part III score was preoperatively evaluated in both medication withdrawal (med‐off) and medication stages (med‐on). Four to five weeks after surgery, the internal pulse generator (IPG) was turned on and programmed. This time, the MDS‐UPDRS III score was evaluated in the stimulation on/medication off stage (stm‐on). The responses to medication and stimulation were calculated as follows^16^:

$$\text{Medication}\;\text{response}=\frac{{\left(\text{MDS UPDRS}\right)}_{\text{med ‐ off}}‐{\left(\text{MDS UPDRS}\right)}_{\text{med ‐ on}}}{{\left(\text{MDS ‐ UPDRS}\right)}_{\text{med ‐ off}}}\times 100\%.$$

$$\text{Stimulation}\;\text{response}=\frac{{\left(\text{MDS UPDRS}\right)}_{\text{med ‐ off}}‐{\left(\text{MDS UPDRS}\right)}_{\text{stm ‐ on}}}{{\left(\text{MDS UPDRS}\right)}_{\text{med ‐ off}}}\times 100\%.$$

All patients were randomly assigned to the training set (*n* = 73) or the test set (*n* = 21).

### 2.2 Neuroimage acquisition

The patients were scanned with 3.0 T MRI scanner (Philips Medical Systems, Best, The Netherlands) with 32‐channel head coil, and the patients’ heads were immobilized accurately with head cushions before surgery. The whole‐head three‐dimensional sagittal T1‐weighted‐3D magnetization‐prepared rapid acquisition gradient echo (MP‐RAGE) sequence (repetition time: 6.6 ms, echo time: 3.1 ms, flip angle: 8°, matrix size: 240 × 240, isotropic voxel: 1 × 1 × 1 mm^3^, number of slices: 196) was acquired from each participant. Also, the T2‐weighted image and fluid‐attenuated inversion recovery were scanned for surgical planning and STN localization. Preoperative (with Leksel frame) and postoperative (the time the IPG was turned on) computed tomography (CT) (thickness: 0.625 mm; General Electric Healthcare, Milwaukee, Wisconsin, USA) was performed.

### 2.3 STN‐DBS surgery

Bilateral STN implantations were performed using a Leksell G frame system with the assistance of a Leksel Surgiplan workstation (Elekta Instrument AB, Stockholm, Sweden) with preoperative MRI and CT. Micro‐electrode recordings and macro‐stimulation were used to accurately target the STN. During electrode implantation, steel cannulas were kept in place. The quadripolar DBS electrodes (47 patients with Model 3389; Medtronic Inc.; 47 patients with Model L301; PINS Medical Co. Ltd.; using the same parameters) were implanted and fixed, and the IPGs were implanted subsequently. After 4–5 weeks, patients were asked to return to the hospital and started the program (in a stable off medication condition), in order to minimize micro‐subthalamotomy effects.^14^, ^17^ Each patient underwent a regular adjustment of stimulation settings, achieving a satisfactory clinical outcome and avoiding intolerable side effects. Then, the motor symptom was measured.

### 2.4 Lead localization and VTA calculation

The lead positions were processed with Lead‐DBS toolbox (Version 2.1.8; www.lead‐dbs.org) based on preoperative MRI and postoperative CT, as described in previous studies.^12^, ^18^, ^19^ Briefly, all DICOM files were converted to NifTi formats. Then, postoperative CT scans were linearly coregistered to the preoperative MRI (MP‐RAGE sequence) using the Advanced Normalization Tools (ANTs; http://stnava.github.io/ANTs/). Registration between postoperative CT and preoperative MRI was further refined with the “brain shift correction” module, which focuses on the subcortical target region of interest and thus minimizes nonlinear bias introduced when opening the skull during surgery.^18^ The images were normalized to standard space (Montreal Neurological Institute [MNI] space) with the symmetric diffeomorphic registration algorithm implemented in the ANTs. Lead trajectories and contacts were automatically pre‐localized with PaCER and manually refined.

In accordance with the stimulation settings of the activated contact, the electric fields around the contact were modeled with a finite element, described in a previous study.^18^ Briefly, an adapted version of the FieldTrip/SimBio pipeline (https://www.mrt.uni‐jena.de/simbio/; http://www.fieldtriptoolbox.org/) was used. All analyses were conducted using the impact of the electric field on the motor STN, whereas measurements were repeated with a binarized version of it. In order to approximate the VTA, a heuristic value of 0.2 V/mm was adapted as a threshold for the electric field gradient magnitude, which has been widely applied in previous studies.^11^, ^12^ The VTA in the present study was taken as the sum of the left and right VTAs (motor STN).

### 2.5 Evaluation of cortical thickness, subcortical structure, and white matter volume

**FIGURE 1:**
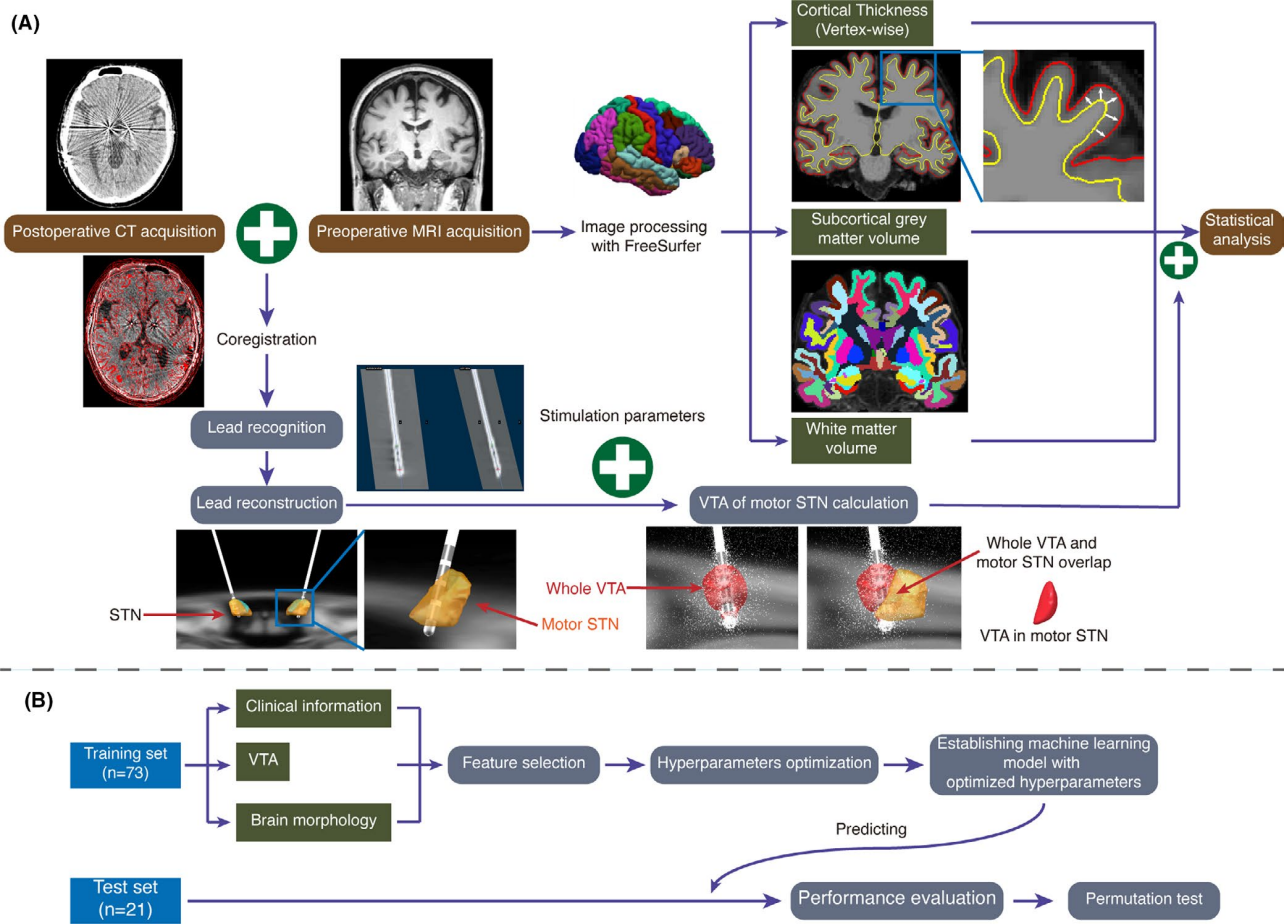
(A) Flowchart of the data processing and statistical analysis. MRIs were processed with FreeSurfer to obtain brain morphology data. The postoperative CT was co‐registered to preoperative MRI and normalized. Lead trajectories and contacts were localized. Then, the VTA in the motor STN was calculated on the basis of program settings and lead position, and it was subsequently used as one of the covariates in the statistical analysis of brain morphology. (B) Flowchart of the stimulation efficacy prediction process on the basis of machine learning. To enhance performance, feature selection and hyperparameter optimization were performed before establishing the final machine learning model, which was then applied to predict the test set. Finally, performance was tested, and the permutation test was performed. CT, computed tomography; MRI, magnetic resonance imaging; STN, subthalamic nucleus; VTA, volume of tissue activated

The preoperative MP‐RAGE images were processed with FreeSurfer (development version, http://www.freesurfer.net) as described previously.^20^ Briefly, the procedures included nonbrain data removal, image intensity normalization, tessellation of the gray/white matter boundary, automated correction of topology, and surface deformation to identify tissue borders. In each vertex, cortical thickness was considered as the distance between the white and gray matter surfaces of the reconstructed cortical mantle. The subcortical structures were also segmented and calculated. The accuracy of segmentation was checked and viewed with FreeView. Based on previous studies, a full width at half maximum of 10 mm was applied to smooth using a circularly symmetric Gaussian kernel across the surface.^21^ The volumes of subcortical gray and white matter were finally adjusted to intracranial volume, referring to a previous study^22^ (Figure 1A).

### 2.6 Automatic prediction with machine learning

The support vector machine (SVM), a supervised machine learning algorithm, can accurately and effectively solve data classification and regression issues.^20^ In the present study, the DBS efficacy prediction was applied with the library for SVM (Version 3.2, https://www.csie.ntu.edu.tw/~cjlin/libsvm/) and MATLAB (Version 2018b, Mathworks, Inc.).^23^

The feature selection technique aims to remove the redundant or irrelevant features or features which are strongly correlated in the data without much loss of information, which could avoid overfitting and enhance performance of the machine learning algorithm.^24^ It should be noted that feature selection was only performed in the training set, avoiding inspection of the test set data. The least absolute shrinkage and selection operator (LASSO), put forward by Tibshirani, has been widely used in feature selection. LASSO transforms each coefficient by a constant component *λ*, truncating at zero.^24^ Based on a previous study, the optimal *λ* with minimum mean square error (MSE) was chosen during cross‐validation (fivefold). Then, the features with nonzero coefficient were considered as the optimal feature set.^25^ The randomly selected hyperparameters used in the SVM could lower the accuracy of prediction; in other words, the performance of the machine learning algorithm could be largely affected by these randomized hyperparameters. Therefore, a genetic algorithm approach, an evolutionary computation algorithm,^26^ was adopted in the present study to obtain optimized hyperparameters (cost and gamma) in machine learning. Finally, the model (radial basis function [RBF] kernel) established with these features and hyperparameters (after scaled) was further used for predicting the test set.

### 2.7 Performance of machine learning evaluation

Pearson correlation was performed to evaluate the correlation between actual and predicted DBS efficacy, obtaining the Pearson correlation coefficient (*r*) and the *p* value of *r*. Also, the coefficient of determination (*R* ^2^) and the MSE in the test set were measured, similar to a previous study.^27^ The permutation test can be used to evaluate the probability of obtaining *R* ^2^ values higher than the ones obtained during test set prediction by chance, which was conducted based on a previous study.^28^ Briefly, the labels were permuted 2000 times. Each time, the labels were randomly assigned to each subject, and the former procedure was repeated, including feature selection, hyperparameter optimization, and prediction. Then, the number of times *R* ^2^ was higher for the permuted labels than for the real labels was counted and dividing this number by the permutation times, we derived a *p* value for the regression (*R* ^2^) (Figure 1B).

### 2.8 Statistical analysis

All data are expressed as mean ± standard deviation or as median (Q1, Q3). The Shapiro–Wilks test was used to evaluate the normality of the data, and non‐parametric tests were used for non‐Gaussian distribution data. The differences in clinical data and basic information between the training and test sets were analyzed using the two‐sample Student *t*‐test, the chi‐squared test, and the Mann–Whitney *U* test. Vertex‐wise analysis was performed to evaluate the association between cortical thickness and DBS efficacy, using a general linear model (GLM) corrected to age, sex, and VTA, followed by Monte Carlo simulation. Similarly, the association between the volume of subcortical gray/white matter and DBS efficacy was measured with the GLM. Nonetheless, results were corrected with a false discovery rate. The statistical analyses were only performed within the training set, avoiding information leak from the test set during prediction. A *p* value < 0.05 was considered to indicate statistical significance.

## 3 RESULTS

### 3.1 Patient demographics

There were 73 patients in the training set and 21 patients in the test set. No significant difference was found with respect to sex, age, disease duration, H‐Y stage, LEDD, ${\text{MDS UPDRS}}_{\text{med ‐ off}}$, ${\text{MDS UPDRS}}_{\text{med ‐ on}}$, ${\text{MDS UPDRS}}_{\text{stm ‐ on}}$, medication response, stimulation response, or VTA in the motor STN between the training and test sets. The details are summarized in Table 1. A significant correlation between medication response and stimulation response was observed (both with [*p* = 0.0306] and without [*p* = 0.0286] correction for VTA), similar to a previous study.^29^

**TABLE 1:** Demographic and clinical details of patients

	Training set (*n* = 73)	Test set (*n* = 21)	*p*‐value
Sex (male/female)	45/28	11/10	0.4459
Age (years, median [Q1, Q3])	63.0 (57.0, 68.3)	65 (61.8, 67.0)	0.5918
Disease duration (years, median [Q1, Q3])	8.0 (5.9, 11.0)	8.0 (6.0, 11.9)	0.4135
H‐Y stage (median [Q1, Q3])	3 (3, 3)	3 (3, 3)	1.0000
LEDD (mg/day, median [Q1, Q3])	698.0 (574.0, 892.5)	799.0 (545.8, 1122.9)	1.0000
MDS‐UPDRSIII (med‐off)	51.0 ± 17.3	50.5 ± 16.7	0.8994
MDS‐UPDRSIII (med‐on)	25.4 ± 15.0	26.0 ± 14.6	0.8707
MDS‐UPDRSIII (stm‐on)	26.2 ± 15.2	27.6 ± 14.1	0.7026
Medication response (%)	51.0 ± 19.1	50.1 ± 21.6	0.8507
Stimulation response (%)	48.0 ± 24.1	45.4 ± 18.8	0.6509
VTA in motor STN (mm^3^)	80.1 ± 49.7	101.6 ± 77.2	0.1298

Abbreviations: H‐Y stage, Hoehn‐Yahr stage; LEDD, levodopa equivalent daily dose; MDS‐UPDRS, Movement Disorder Society Unified Parkinson's Disease Rating Scale; med, medication; stm, stimulation; STN, subthalamic nucleus; VTA, volume of tissue activated.

### 3.2 Association between cortical thickness and stimulation improvement

**FIGURE 2:**
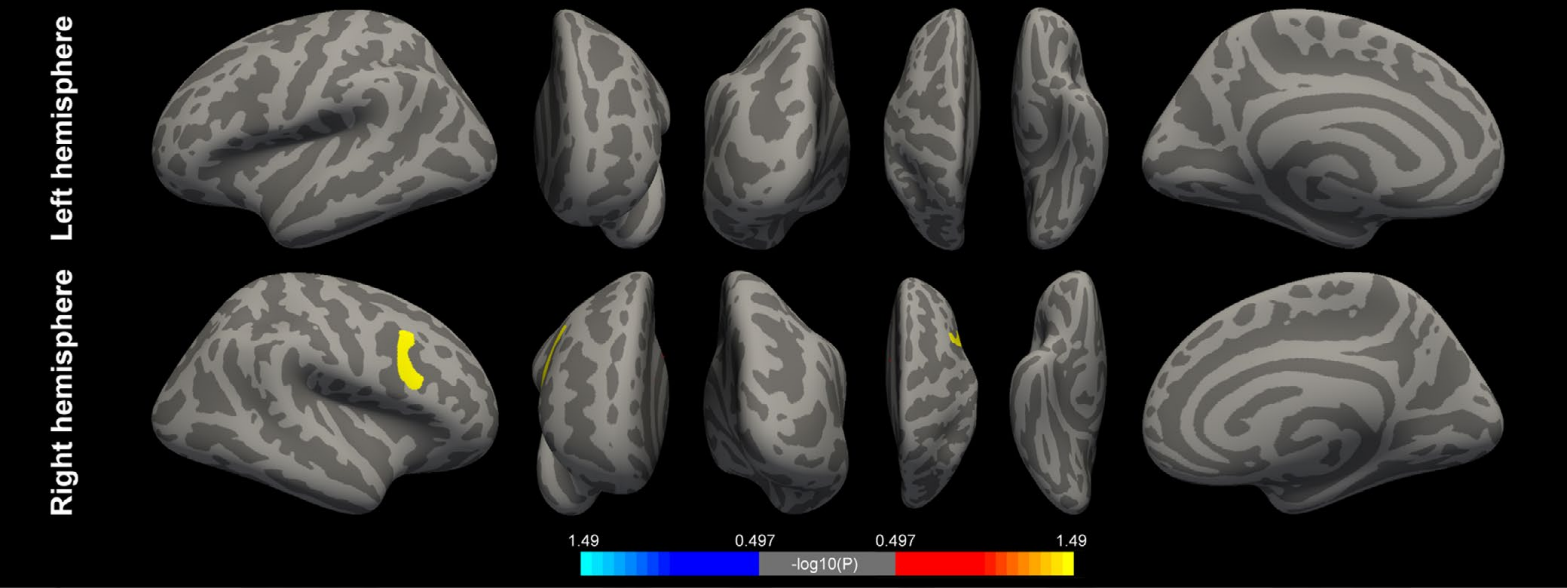
Vertex‐wise analysis of the association between cortical thickness and stimulation efficacy. The right precentral cortical thickness (yellow cluster) is positively associated with initial stimulation‐related motor improvement. Teal/blue, negative correlation; red/yellow, positive correlation

In accordance with a previous study, the motor improvement in response to stimulation was associated with VTA; therefore, VTA needs to be considered during statistical analysis.^12^ Considering the MDS‐UPDRS III score reflects the severity of bilateral motor symptoms of PD, the VTA used in the GLM was the sum of the left and right VTAs. We observed that the right precentral inferior region was significantly associated with the stimulation‐related motor improvement (Figure 2). The details of the cluster are shown in Table 2. Additionally, our results indicate that the PD patients with thicker right precentral inferior cortices were more likely to achieve better STN stimulation‐related motor improvements.

**TABLE 2:** Association of the cortical thickness with DBS improvement

	Cortical area	Cluster size (mm^2^)	Cluster‐wise *p*‐value	MNI coordinates (mm)		
				*x*	*y*	*z*
ROI1	Right precentral inferior part	454.7	0.0343	37.7	10.6	22.5

Note

Monte Carlo simulation.

Abbreviations: DBS, deep brain stimulation; MNI, Montreal Neurological Institute; ROI, region of interest.

### 3.3 Association between the volume of the subcortical gray and white matter and stimulation improvement

The GLM was applied to investigate the association between gray/white matter volume and the stimulation response. Though some white matter volumes were found to be associated with motor improvements after stimulation, no statistical significance of these regions was observed after false discovery rate correction. There is no association between the volume of the subcortical gray structure and stimulation‐related motor improvement, both with and without correction.

### 3.4 Individual prediction of stimulation improvement

To evaluate the performance of the individual prediction of stimulation improvement and choose the optimal feature set, machine learning was conducted using different feature sets.

**FIGURE 3:**
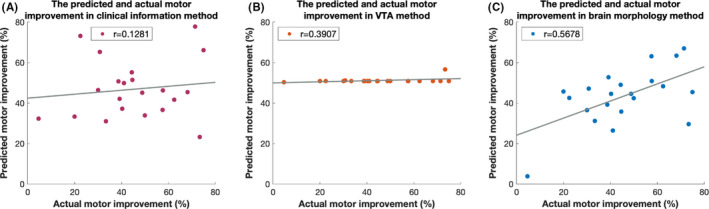
(A–C) Correlation between actual and predicted stimulation efficacy using methods based on different feature sets. The performance of the machine learning algorithm using the brain morphology method (*r* = 0.5678, *p* = 0.0073) was much better than that using the clinical information (*r* = 0.1281, *p* = 0.5801) and VTA (*r* = 0.3907, *p* = 0.0799) methods

**FIGURE 4:**
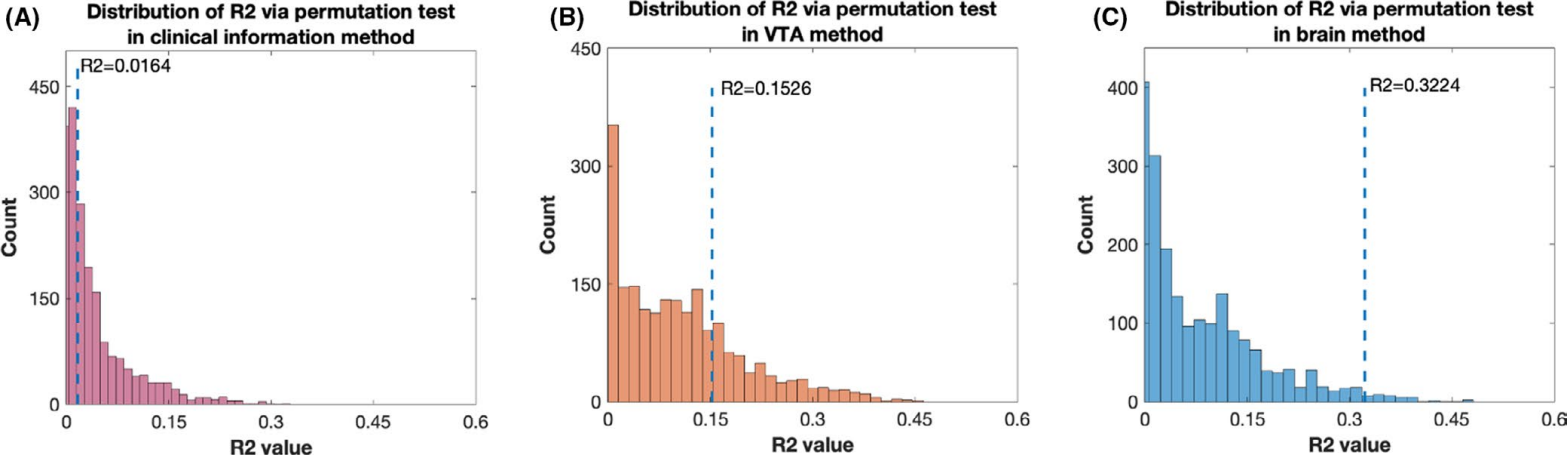
(A–C) The permutation test of machine learning (*R* ^2^ distribution) using methods based on different feature sets. The performance of the machine learning algorithm using the brain morphology method was not based on chance (*p* = 0.0185); however, the performance using the clinical information (*p* = 0.5725) and VTA (*p* = 0.2645) methods might be

Firstly, clinical information, including sex, age, and medication response, was used to predict STN‐DBS‐related motor improvement (clinical information method). Disappointingly, a very low predictive value, with an *r* value of 0.1281 and an *R* ^2^ of 0.0164, was observed (Figure 3A). The results of the permutation test indicated the prediction of the stimulation response may be purely based on chance (Figure 4A). Then, we added VTA data to the clinical information to establish a new model (VTA method). The results of this model were still unsatisfactory, with an *r* value of 0.3907 and an *R* ^2^ of 0.1526 (Figure 3B). A lower *p* value was obtained by the permutation test, but statistical significance was not achieved (Figure 4B). The detailed results of the machine learning algorithm are shown in Table 3 and Table S1. Moreover, we used the multiple linear model with clinical information and VTA data to predict the stimulation response. A much worse result (*r* = −0.1797, *R* ^2^ = 0.0323) was obtained compared with that of SVM (Figure S1).

**TABLE 3:** The performance of the machine learning algorithm on initial DBS efficacy

	Clinical information method	VTA method	Brain morphology method
Features	Sex, age, medication response	Sex, age, medication response	Sex, age, medication response
		VTA in motor STN	VTA in motor STN
			Brain morphology
*r*	0.1281	0.3907	0.5678
*p* value of *r*	0.5801	0.0799	0.0073
*R* ^2^	0.0164	0.1526	0.3224
*p* value of permutation test (for *R* ^2^)	0.5725	0.2645	0.0185

Abbreviations: *r*, Pearson correlation coefficient; *R* ^2^, coefficient of determination; VTA, volume of tissue activated.

However, a much more satisfactory and exciting result of the SVM was obtained using the feature set which combined clinical information, VTA, and brain morphology (the brain morphology method). A high correlation of actual and predicted stimulation improvement was observed, with an *r* value of 0.5678 and an *R* ^2^ of 0.3224, indicating a potential clinical application value (Figure 3C). Subsequently, the permutation test revealed that the results predicted with the present model were unlikely to be based purely on chance (Figure 4C). The details are summarized in Table 3 and Table S1. Furthermore, based on a previous study, the average error deviation was also calculated.^11^ The predictions using this model deviated on average by 11.4 ± 10.9% from the actual MDS‐UPDRS III stimulation response. For instance, if a patient actually improved by 50%, our algorithm might predict an improvement between 61.4% and 38.6%.

## 4 DISCUSSION

The present study (i) illustrates the association between brain morphology and initial STN‐DBS efficacy for motor improvement and (ii) predicts the efficacy using these features and machine learning. We demonstrated that the cortical thickness of the inferior part of the right precentral cortex was associated with STN‐DBS efficacy. Using brain morphological features, STN‐DBS efficacy could be predicted with an *r* value of 0.5678, indicating a potential clinical application value.

### 4.1 Brain morphology and network changes in PD and association with DBS

Brain morphology and network changes have been found to be related to PD pathology and associated with motor symptoms.^8^, ^9^, ^30^ In the present study, the right precentral cortical thickness was found to be positively correlated with stimulation‐related motor improvement. The precentral gyrus belongs to the motor cortex and is the origin of the pyramidal tract. Atrophy of the precentral cortex in PD has been observed previously.^31^, ^32^ Additionally, PD patients showed significantly weaker brain activation in the precentral cortex, putamen, frontal gyrus, and thalamus. Nevertheless, PD patients who underwent acupuncture, an additional treatment for relieving PD symptoms, achieved stronger activation of the precentral cortex, frontal gyrus, and putamen, as observed by fMRI.^33^ In a positron emission tomography study, in the “on” condition, PD patients with levodopa‐induced dyskinesias exhibited higher 11 C‐CNS 5161 (a marker of activated N‐methyl‐D‐aspartate receptor ion channels) uptake in the precentral gyrus, caudate, and putamen compared with patients without dyskinesias, indicating that dyskinetic patients might have abnormal glutamatergic transmission in motor areas.^34^ Therefore, the impairment of the precentral gyrus may contribute to the motor deficits.

DBS has been proved to be both beneficial to the motor symptoms and nonmotor symptom (such as cognitive function).^35^, ^36^ Some studies further investigated the changes related to DBS. Kahan et al. used fMRI to investigate the alterations of indirect, direct, and hyper‐direct pathways of the basal ganglia‐cortical loops under STN‐DBS using dynamic causal modeling and found it could strengthen cortico‐striatal and thalamocortical pathways.^37^ However, the position of lead and VTA were not considered in this study. Therefore, a more recent PD study took these issues into account and showed that the relationship between STN‐DBS placement and the increase in connectivity in the motor network, which was accompanied by an increase in coupling between the motor thalamus and the motor cortex, was strong. Moreover, the more optimally an STN‐DBS electrode was located (as measured by VTA in the motor STN), the more normal the overall functional connectivity became.^12^

Undoubtedly, the DBS position could largely affect the stimulation‐related motor improvement. At one extreme, if the leads are far away from the target, it is hard to achieve an ideal therapeutic efficacy. Considering the recommendations from a former study,^12^ VTA was used as a covariate. Therefore, we believe that our results are convincing. Our observations that a thicker right precentral cortex is associated with a stronger motor response to initial STN‐DBS improve our understanding of the mechanisms underlying the positive effects of STN‐DBS.

### 4.2 Predicting DBS efficacy with machine learning

Machine learning is based on algorithms that can learn from data and predict characteristics of another data set, without relying on rule‐based programming.

One previous study classified DBS efficacy into three categories and predicted it using clinical information, lead position, and program settings, obtaining an accuracy of 86% (12/14).^38^ Although the performances of the different methods were tremendously different in our study, similar accuracies (95.2%, 81.0%, and 95.2% using the clinical information, VTA, and brain morphology methods, respectively) were obtained. Therefore, we believe that *r* and *R* ^2^ are more sensitive than accuracy to evaluate the performance of DBS efficacy prediction. One study reported that DBS efficacy was associated with medication efficacy,^16^ whereas another study contradicted these results,^11^ which convinces us that the feature set including only clinical information and VTA data may not achieve an acceptable performance.^39^ Hence, we added the brain morphological features to the machine learning model and achieved a much more better prediction performance. We speculate that in some patients with specific brain morphological features, medication response could be an ideal predictor of medication improvement; however, in other situations, it may not be an ideal predictor.

Some other studies only showed which factors may have a predictive value; however, they were not applied in the prediction with the “test set”.^16^, ^40^ These studies highlight promising research directions to improve DBS efficacy prediction, whereas future experiments using these features to predict DBS efficacy in the “test set” need to be performed. However, some studies have also predicted the DBS efficacy in a “test set.” Horn et al. combined publicly available human connectome data (DTI and resting fMRI) and applied these features to predict outcome in the “test set”. DTI (fiber connectivities) and resting fMRI (functional connectivities) were independent predictors of (long‐term) stimulation improvement and could be used to predict the motor improvement response in individual patients with an average error of 15% (*r* = 0.34 for DTI; *r* = 0.45 for fMRI).^11^ Our results are better than those previously reported, which may be attributed to the use of publicly available human connectome data (instead of “training set” connectome data), different brain features (morphology in our study), and different DBS terms. Gonzalez‐Escamilla et al. classified dystonia patients who underwent globus pallidus internus‐DBS in the superior‐outcome group or the moderate‐outcome group based on DBS efficacy, and the brain morphology network fingerprints were used to predict DBS efficacy, achieving an accuracy of 88%.^20^

The long‐term programming is based on the initial programming, and the long‐term DBS motor response is closely related to the initial efficacy. However, some differences between initial and long‐term DBS issues still exist.^14^ Some studies investigated the association between DBS long‐term efficacy and the brain network,^11^ but the association between brain morphology and the initial DBS motor response has not been well illustrated previously. Some patients could benefit more from aDBS, additionally, with less electric consumption to some degree, aDBS is like a continuous “on/off” switch. Therefore, investigations of initial stimulation efficacy‐related factors and their predictive values may contribute to the development of aDBS. However, the control of aDBS is mainly based the biomarkers, such as electrophysiological measurements, neurochemical sensing, and external mechanistic sensors.^41^ As the disease progresses, the cortical pattern may be changed.^6^ Hence, further studies that evaluate the value of our results in aDBS need to be performed. Moreover, macro‐stimulation is conventionally conducted during surgery to guarantee benefits of the surgery to the motor symptoms.^42^ If initial DBS efficacy could be accurately predicted, this procedure may could be omitted.

There are some limitations in our study. Firstly, the size of the cohort was not very large. Although other studies focusing on the DBS outcome only involved a small number of patients,^11^, ^20^ there is no doubt that the large cohort could enhance the reliable of model. In the future, a multi‐center study with a larger sample size may extend the application value of the present study. Secondly, the pathological change of PD is not only related to structural alternation, the metabolism and other function changes are also involved in pathology. Dopaminergic dysfunction and abnormal metabolic activity have been observed in PD patients and play an indispensable role in pathogenesis of PD. Predicting the outcome of DBS with the additional features, including cerebral glucose and dopamine metabolism observed via positron emission tomography, may enhance the efficacy of the model.^43^, ^44^ As mechanism of STN‐DBS being more profound and extensive, DBS settings may be automatically programmed, and perhaps, accurate prediction of DBS efficacy may be achieved in the future based on only lead position, the cerebral structural and function change (such as structural MRI, positron emission tomography and functional MRI), without the need for VTA data.

## 5 CONCLUSION

We found that the right precentral cortical thickness is positively associated with initial stimulation‐related motor improvement; however, similar results for the volume of the subcortical gray or white matter were not observed. The potential clinical value of predictions of initial STN‐DBS efficacy was obtained when using brain morphology features, with an *r* of 0.5678 and an *R* ^2^ of 0.3224. These predictions on average deviated by 11.4 ± 10.9% from actual improvements.

## CONFLICTS OF INTEREST

None.

## AUTHOR CONTRIBUTIONS

Yingchuan Chen, Guanyu Zhu, Defeng Liu, Yuye Liu, Tianshuo Yuan and Xin Zhang were involved in data collection. Yingchuan Chen, Guanyu Zhu, Yin Jiang, Tingting Du and Jianguo Zhang were involved in data analysis. Jianguo Zhang conducted the surgery and designed the study.

## Supporting information

Fig S1

Table S1

## Data Availability

The data that support the findings of this study are available from the corresponding author upon reasonable request.
