# Supplementary figures and images for: Predict initial subthalamic nucleus stimulation outcome in Parkinson's disease with brain morphology
Source: CNS Neurosci Ther. 2022 Jan 20;28(5):667–76. doi: 10.1111/cns.13797 (PMC8981473; doi:10.1111/cns.13797)

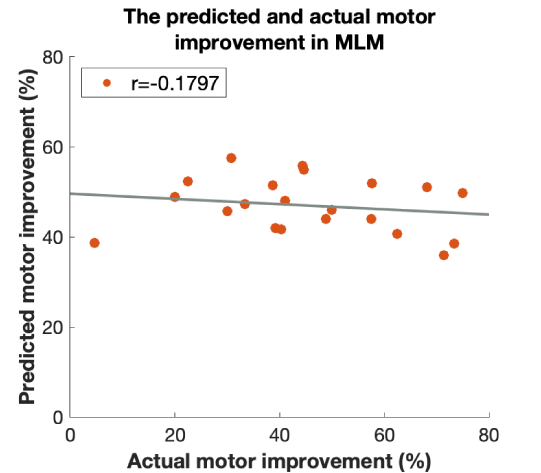

Supplement: Supplementary file 1 — Fig S1 [file CNS-28-667-s001.tif]
