# Supplementary material for: Predict initial subthalamic nucleus stimulation outcome in Parkinson's disease with brain morphology
Source: CNS Neurosci Ther. 2022 Jan 20;28(5):667–76. doi: 10.1111/cns.13797 (PMC8981473; doi:10.1111/cns.13797)
Supplement: Supplementary file 2 — Table S1 [file CNS-28-667-s002.docx]

**Supplementary Table 1.** The detailed information of machine learning.

|  | **Clinical information method** | **VTA method** | **Brain morphology method** |
| --- | --- | --- | --- |
| Brain morphology feature | － | － | ROI of cortical thickness |
|  |  |  | White matter: right cuneus, right inferiortemporal, right lateraloccipital, right postcentral |
| MSE | 0.0467 | 0.0352 | 0.0242 |
| Cost | 43.8 | 9.9 | 59.8 |
| Gamma | 5.3 | 1000 | 0.2 |

MSE, mean square error in the test set.
